# Bisphenol A and cancer: a prelude to challenging epidemiology

**DOI:** 10.1007/s00420-021-01752-5

**Published:** 2021-08-27

**Authors:** Thomas C. Erren

**Affiliations:** Institute and Policlinic for Occupational Medicine, Environmental Medicine and Prevention Research, Faculty of Medicine and University Hospital Cologne, University of Cologne, Kerpener Str. 61, 50938 Cologne, Germany

**Keywords:** Bisphenol, Cancer, Dose, Timing, Perinatal, Adult, Dose–response relationship

To the Editor,

López‑Carrillo and colleagues contributed case–control analyses on possible links between an important “plastics” ingredient and cancer (Lopez-Carrillo et al. 2021). The authors emphasize that “this first report” on free-bisphenol A (BPA-F) and breast cancer in women—with an increased odds ratio for the highest category of urinary BPA-F concentrations—needs replication. A recent meta-analysis (Liu et al. 2021) documented “no association” between BPA and breast cancer. Importantly, the publications discussed critical questions regarding BPA exposures and that the field needs better designed high-quality epidemiology. To this end, this letter complements considerations regarding (i) biological plausibility, (ii) dose and timing, and (iii) dose–response relationships when researching facets of a mindboggling complexity around BPA (Benno Meyer-Rochow et al. 2015; Huff 2003; Seachrist et al. 2016).

Regarding (i), still one decade ago, the WHO concluded that “…there is currently insufficient evidence on which to judge the carcinogenic potential (of bisphenol A)” (WHO 2010). In the meantime, accumulated evidence from rodent studies suggests that early-life BPA exposures below the oral reference dose established in 1982 (National Toxicology 1982) may lead to increased cancer susceptibility (mammary and prostate) (Benno Meyer-Rochow et al. 2015; Seachrist et al. 2016). IARC has not yet classified bisphenol A but an advisory group recommended such review as a high priority (IARC 2014).

Regarding (ii), Paracelsus paved the road to toxicology with “What is there that is not poison? All thing﻿s are poison and nothing is without poison. Solely the dose determines that a thing is not a poison” (Grandjean 2016). His sixteenth century dictum “the dose makes the poison” should be extended for the new Developmental Origins of Health and Disease (DOHaD) (Heindel and Vandenberg 2015) paradigm with “the timing makes the poison” (Grandjean et al. 2008). To exemplify, if different time windows render us differentially susceptible to different doses, much lower BPA doses may be detrimental when experienced in and/or ex-utero than those which we may consider “safe” for adults.

Regarding (iii), studies of large occupational cohorts in the plastics industry contributed to IARC’s classification of synthetic plastic polymers as carcinogenic (vinyl chloride: Group 1) (Baan et al. 2009) or probably carcinogenic (styrenes: Group 2A) (IARC Monographs Vol 121 Group 2018) to humans. But beyond such “classical” studies in adults, in regards to BPA, we may want to look at “novel”, viz newly appreciated, dose–response relationships as well—or in particular (Benno Meyer-Rochow et al. 2015). In animal experiments, both BPA’s carcinogenic (Soto and Sonnenschein 2010) and epigenomic disruption (Bernal and Jirtle 2010) properties have been demonstrated: epidemiologists should thus explore whether doses with no discernible effects in adults may affect development in and/or ex-utero which could predispose individuals to cancer later in life (Benno Meyer-Rochow et al. 2015).

Overall, considering (i) and (ii) and (iii), the above study (Lopez-Carrillo et al. 2021) can be but a prelude to much-needed and very complex research—no less and no more. We should not assume that BPA exposures are constant over time and that a single urine sample is representative over time (Lopez-Carrillo et al. 2021). Neither should we ignore the possibility that different doses in different time windows are associated with differential cancer risks. While presenting epidemiology with significant challenges, to avoid dose–response fallacies, interpretable research regarding BPA and cancer should be based on valid—preferably longitudinal—assessments of both early and adult-life BPA exposures (Liu et al. 2021).
